# Dublin Anti-Bullying Self-Efficacy Models and Scales: Development and Validation

**DOI:** 10.1177/08862605221127193

**Published:** 2022-09-30

**Authors:** Aikaterini Sargioti, Seffetullah Kuldas, Mairéad Foody, Paloma Viejo Otero, Angela Kinahan, Colm Canning, Darran Heaney, James O’Higgins Norman

**Affiliations:** 1 DCU Anti-Bullying Centre, Dublin City University, Dublin, Ireland; 2 Department of Media and Communication, University of Oslo, Oslo, Norway; 3 School of Psychology, National University of Ireland, Galway, Ireland

**Keywords:** victim, bystander, self-efficacy scale, offline bullying, cyberbullying, scale development, scale validation

## Abstract

Literature on anti-bullying programs shows a growing consensus about promoting victims and bystanders’ self-efficacy against bullying, but provides no theoretical model nor measurement scale to assess the extent of achieving this aim. The current research aims to address these theoretical and empirical gaps by proposing the Dublin Anti-Bullying Self-Efficacy Models and Scales, using a convenience sample of 14-year-old students in Ireland (*N* = 1,100). After establishing both content and face validity, four separate scales were tested to measure anti-bullying self-efficacy beliefs among offline victims (20-item), online victims (20-item), offline bystanders (20-item), and online bystanders (20-item). Thereafter, four separate exploratory factor analyses of the scale items were followed by reflective measurement analyses of their internal consistency and construct (convergent and discriminant) validity. Results indicated sufficient psychometric properties of each scale measuring five dimensions of anti-bullying self-efficacy: recognition, emergency comprehension, responsibility, knowledge, and intervention. Further research is needed to test the proposed model and scale for assessing effectiveness of an anti-bullying program in promoting self-efficacy beliefs.

To be a target of peer-bullying and cyberbullying is a continuous cause for worry among students, teachers, and parents around the world (Salmivalli et al., 2021). Children and adolescents who have been victims of offline or online bullying may suffer from mental health and/or psychosomatic problems, such as depression, poor self-esteem, anxiety, suicidal ideation and/or headaches and sleep disturbances, respectively (Olweus, 2012). To prevent or reduce bullying behaviors and the consequences, a growing consensus suggests that anti-bullying programs should have a particular focus on promoting anti-bullying self-efficacy beliefs among victims (Salimi et al., 2021), bystanders (Green et al., 2020; Thornberg et al., 2017; Wachs et al., 2018), or both (Andreou et al., 2007; O’Moore & Minton, 2005). Anti-bullying self-efficacy beliefs of victims and bystanders can have a preventative impact on bullying and cyberbullying behaviors.

However, due to an absence of scales measuring effectiveness of anti-bullying programs in terms of promoting victim and bystander’s self-efficacy in tackling bullying and cyberbullying behaviors, the consensus falls short of empirical evidence. There are theoretical approaches and models, namely the *Participant Role Approach* (Salmivalli et al., 1996) and *Bystander Intervention Model* (Latané & Darley, 1970), which support the consensus about self-efficacy development. However, these theoretical perspectives have yet to be synthesized for identification and measurement of various dimensions of victim and bystander’s self-efficacy. To date, there is no scale which aims to measure the extent to which victims and bystanders have confidence in their ability to tackle bullying and cyberbullying behaviors. This presents an empirical gap in the literature on anti-bullying programs, which for decades, has attempted to empower students to prevent and reduce bullying at school.

To address this empirical gap, the present research synthesizes the participant role approach and bystander intervention model, and thus, proposes *a Social-Ecological Approach and Model of Anti-Bullying Self-Efficacy*. This proposed approach allows for the identification of self-efficacy dimensions, referred to as five steps that victims and bystanders take to intervene in online and offline bullying behaviors. These five steps are defined as victim and bystander’s self-efficacy to: (1) *recognize* bullying behavior, (2) *comprehend* the need of *emergency* for stopping aggressive behavior, (3) take *responsibility* to intervene in or tackle bullying behavior, (4) *know* what to do, and (5) *intervene* in bullying behavior by reporting or taking actions. The present research is hereby aimed at developing and testing four *separate* scales of the victim and bystander’s self-efficacy, measuring the five dimensions in tackling offline bullying separately from online bullying.

The present paper is comprised of six main sections. First, it presents a critical overview of the traditional and revised definitions of bullying and cyberbullying, and thus, argues how they are interconnected, yet distinct phenomena for measurement purposes. This argument elaborates on why self-efficacy in tackling offline bullying can be measured separately from online bullying using separate scales for: (1) offline victim’s self-efficacy, (2) online victim’s self-efficacy, (3) offline bystander’s self-efficacy, and (4) online bystander’s self-efficacy. Second, it introduces the social-ecological approach and model of anti-bullying self-efficacy for the identification of self-efficacy beliefs among victims and bystanders of offline and online bullying. Third, it provides a rationale for the present study. Fourth, it includes the research methods, measures, and data analyses of the present research. Fifth, it shows the results for content, face, and construct validity, and reliability of the four scales. Last, it discusses results, limitations, and recommendations for further research.

## Overlap and Difference Between Bullying and Cyberbullying

Bullying is traditionally defined as a proactive aggressive behavior having three characteristics; bullying behavior is (a) intentional to harm or hurt a person or group, (b) repetitive (more than twice), and (c) carried out by an individual or a group who are more powerful than the targeted person (Olweus, 1999). Bullying behaviors can be physical, verbal, and social/relational and target sexuality, ethnicity, religion, or (dis)ability of a person or group (Foody et al., 2017). The increasing use of digital technology and the Internet has created a means for *cyberbullying* (Pichel et al., 2021), defined as “willful and repeated harm inflicted through computers, cell phones, and other electronic devices” (Hinduja & Patchin 2015, p. 11). This means students can be involved in both offline and online bullying concurrently. The same person can be a victim, perpetrator, or bystander in both phenomena; also, a victim of offline bullying can become a perpetrator of cyberbullying, or vice versa (Temko, 2019). Therefore, recommendations of anti-bullying programs often assume that offline bullying and cyberbullying are overlapped by definition (Palladino et al., 2016; Pichel et al., 2021).

However, there is still no consensus on the difference or overlap between offline bullying and cyberbullying at the process level (Menin et al., 2021; Pichel et al., 2021; Sticca & Perren, 2013; UNESCO, 2020; Waasdorp & Bradshaw, 2015). Although both cyberbullying and offline bullying may co-occur (Pichel et al., 2021) and have similar negative effects or consequences (Kubiszewski et al., 2015; Waasdorp & Bradshaw, 2015), evidence falls short of being conclusive as to whether bullying and cyberbullying share common conceptual and theoretical characteristics (Dooley, 2009; Pichel et al., 2021; Sabella et al., 2013). Olweus (2012) argued that cyberbullying is merely a form of traditional bullying. In contrast, further evidence suggests that cyberbullying is a conceptually distinct social-technological phenomenon (Kubiszewski et al., 2015; Lazuras et al., 2017; Menin et al., 2021; Sabella et al., 2013).

Albeit offline bullying and cyberbullying have significant correlations in some cases (Lazuras et al., 2017), all the defining criteria of offline bullying (i.e., power imbalance, repetitiveness, intentionality, and negative effects) are not applicable as defining characteristics of cyberbullying (Menin et al., 2021; Waasdorp & Bradshaw, 2015). As the most salient form, the criterion for defining physical forms of offline bullying (e.g., punching, kicking, slapping, or pushing) are not applicable to cyberbullying. In particular, the criteria of power imbalance and repetitiveness distinctively manifest themselves in digital technology with no restriction on the day, time, and place. For example, a victim of offline bullying can become a perpetrator of cyberbullying or the reverse (Cosma et al., 2020). Hence, the shift from online to offline bullying/victimization due to the changes in (a) power-imbalance, (b) day-time-place, and (c) repetitiveness can be considered as three unique characteristics of cyberbullying/cyber-victimization.

First, one of the main conceptual flaws in considering cyberbullying as indistinct to offline bullying arises from the power imbalance criterion. Unlike offline bullying, the conceptualization of power imbalance in cyberbullying involves no physical/face-to-face power but relies on digital skills (Dooley, 2009; Knauf et al., 2018; Kubiszewski et al., 2015; Lazuras et al., 2017; Menin et al., 2021; Waasdorp & Bradshaw, 2015). In some cases, online anonymity may render perpetrators more powerful than victims, and thus, makes victims of cyberbullying feel unable to control the aggressive behavior (Knauf et al., 2018).

Next, the inability of control or feeling powerless can also emerge from day-time-place characteristics of cyberbullying (Dooley, 2009). Offline bullying happens on a specific day, time, and place where victim and perpetrator face each other in person (Kubiszewski et al., 2015). In contrast, cyberbullying/cyber-victimization can happen at any day, any time, and anywhere.

Last, the criterion of repetitiveness is not necessarily a characteristic of cyberbullying. Repetitiveness for cyberbullying may refer to redistribution or re-emergence of a once-off incident of an online aggressive behavior against a person or group (O’Moore, 2014). Therefore, an online offensive post as a once-off incident can re-emerge or be re-shared, and thus, victimize a person or group more than once (Baldry et al., 2017; Dooley, 2009; Knauf et al., 2018; Kubiszewski et al., 2015; O’Moore, 2014; Waasdorp & Bradshaw, 2015).

It is worth noting that, UNESCO (2020) has proposed the exclusion of repetitiveness and intentionality criteria from the traditional definition, proposing an overlap between offline and online bullying characteristics. According to the new definition by UNESCO (2020), school bullying can (a) happen in-person and online within a social network, (b) cause physical, emotional, or social harm to targeted students, and (c) be characterized by an imbalance of power; school bullying can be empowered or disempowered by social, school, and institutional norms or systems. Although this new definition postulates a degree of overlap between online and offline bullying characteristics, it does not mean that self-efficacy beliefs in tackling online and offline bullying also overlap. For example, self-efficacy in tackling offline bullying when being punched, slapped, or kicked can change when bullied online with slur comments on race, ethnicity, or sexual orientation. Such differences in power-imbalance characteristics of both phenomena warrant measuring both victim and bystander’s self-efficacy in tackling offline bullying separately from online bullying behaviors.

## A Social-Ecological Approach and Model of Anti-Bullying Self-Efficacy

The concept of self-efficacy has several distinct or contradictory definitions. The most commonly used definition was made by Bandura, who defined self-efficacy as the belief in someone’s ability to carry out a specific behavior in a successful way (Bandura, 1997). However, this definition considers self-efficacy as an individual trait, and thus, lacks the account of social-ecological effects (Carey & Forsyth, 2009) on the individual’s ability in tackling or dealing with a bullying incident (Kuldas & Foody, 2022). Beliefs in one’s ability to prevent or intervene in a bullying situation are affected by dynamic interactions between individual and social-ecological characteristics, such as student-teacher, child-parent, peer-to-peer interactions (Andreou et al., 2007; Kuldas & Foody, 2022). Effects of person-environment interactions can vary depending on the social-ecological context, such as school anti-bullying policy, teacher’s self-efficacy and attitudes, classroom ethnic composition, and parental self-efficacy (Kuldas & Foody, 2022). This suggests approaching self-efficacy from the social-ecological perspective, taking into account various effects of social-ecological characteristics (Carey & Forsyth, 2009). The present study adopts the social-ecological approach (Kuldas & Foody, 2022) and defines self-efficacy beliefs as a mixture of individual and social-ecological characteristics; self-efficacy is the developmental capacity, process, and outcome of person-environment interactions. When victims or bystanders have a caring and supportive teacher, parent, and/or friend, they can develop and demonstrate self-efficacy in tackling incidents of bullying (Kuldas & Foody, 2022).

Similar to self-efficacy, while certain individual characteristics (*psychological aspect*) can be associated with bullying behaviors, social characteristics (*sociological aspect*) are central to approval/disapproval of a school environment (*educational aspect*) where a bullying incident takes place (O’Higgins Norman, 2020). Therefore, both offline and online bullying need to be considered social rather than individual phenomena, which comprise interpersonal aspects as victim, perpetrator, and bystander (Green et al., 2020; Kokkinos & Kipritsi, 2011; Palladino et al., 2016; Protogerou & Flisher, 2012). When these social phenomena take place, behaviors of a victim and perpetrator are directly distinguishable, but bystanders can have different behaviors or roles, which make them central to anti-bullying programs (Green et al., 2020; Knauf et al., 2018; Salmivalli et al., 2005). To understand bystander behaviors, the *Participant Role Approach* (Salmivalli et al., 1996) and *Bystander Intervention Model* (Latané & Darley, 1970) provide significant insights.

The participant role approach suggests distinguishing between four bystander roles, which are assistant, reinforcer, outsider, and defender (Andreou et al., 2007; Salmivalli et al., 1996). While personal and social-environmental reasons for the assistant and reinforcer roles are largely unclear, one reason for the outsider role is the *diffusion of responsibility* (Latané & Darley, 1970), diffusing personal responsibility to intervene in or stop bullying behavior, mainly because of the perception that someone else will intervene (Thornberg & Jungert, 2013; Wachs et al., 2018). Other individual reasons can be a lack of ability or knowledge to: (a) recognize bullying behavior, (b) realize the emergency situation, and (c) be acquainted with how to tackle bullying behavior (Andreou et al., 2007). Therefore, the participant role approach suggests anti-bullying programs include three steps: (a) *raising awareness*, whereby students need to understand what bullying is and how it feels to be bullied, (b) *self-reflection*, where students reflect on their own knowledge and behavior regarding bullying situations, and (c) *commitment to anti-bullying behaviors*, where students need to learn about their ways or actions to stop bullying either as an individual or group (Salmivalli et al., 2005).

However, the participant role approach lacks clarity of the sequence and number of steps, such as whether self-confidence to intervene in bullying behavior precedes or succeeds self-awareness of that behavior. Victim and bystander’s awareness of both bullying behavior and the need to stop it is not sufficient to tackle a bullying incident (Wachs et al., 2018). Victims and bystanders also need to have confidence in their ability to tackle bullying behavior (Thornberg & Jungert, 2013; Thornberg et al., 2017; Wachs et al., 2018).

Latané and Darley’s (1970) early study on bystander roles provides further insights into the sequence and number of steps. They proposed the bystander intervention model to explain why bystanders choose or do not choose to intervene in aggressive behavior. The model was later adapted for understating bystander roles in bullying behaviors (see Knauf et al., 2018; Nickerson et al., 2014). According to the intervention model (Knauf et al., 2018; Latané & Darley, 1970; Nickerson et al., 2014), bystanders may first, *notice* the incident, and then, *interpret* it as emergency, and last, decide on personal *responsibility* to act; they may further *know* how to intervene (choosing a way to intervene) and implement that *intervention* method. Therefore, effectiveness of an anti-bullying program may be assessed by developing and measuring the bystander ability pertaining to each step (Nickerson et al., 2014).

## The Present Study

Many established anti-bullying programs are aimed at the prevention and/or intervention of victimization and bullying behaviors. To achieve this aim in the school context in Ireland, such programs are largely focused on raising awareness, implementing anti-bullying policies, and promoting a positive school climate (Foody et al., 2018), and therefore, their effectiveness is generally assessed in terms of achievement of these aims. Such an assessment method lacks a central focus on the development and measurement of victim and bystander’s self-efficacy, which play a central role in the prevention and intervention of offline bullying and cyberbullying behaviors (Salimi et al., 2021). Therefore, anti-bullying programs can be more effective in the prevention of victimization through the enhancement and measurement of victim and bystander’s self-efficacy (Green et al., 2020; Nocentini & Menesini, 2016; Salimi et al., 2021; Thornberg et al., 2017).

Assessments of effectiveness of school anti-bullying programs are usually based on students’ self-reports of the victimization/bullying incidents after the program implementation. If students’ self-reported incidents are higher after or lower prior to the anti-bullying program, it may be considered ineffective (Minton et al., 2013; O’Moore & Minton, 2004, 2005). However, such an assessment or conclusion can be misleading because the higher rate can be a result of raised awareness about bullying behaviors rather than an actual increase in bullying incidents. In other words, the higher rate does not mean *ineffectiveness*, but *effectiveness*, of an anti-bullying program in promoting victim and bystander’s self-efficacy. Hence, an accurate assessment of effectiveness of school anti-bullying programs needs to account for students’ self-efficacy in tackling bullying behaviors (Garandeau et al., 2014; Kuldas & Foody, 2022).

School anti-bullying programs need to have a particular focus on increasing victim and bystander’s confidence in their ability to tackle bullying behavior in a legally acceptable and safe way (O’Moore & Minton, 2005). However, there is a scarcity of published research on the measurement of both victim and bystander’s self-efficacy in bullying situations across countries, including Ireland. Our literature review found only one study (see Andreou et al., 2007) that addressed the need for an assessment of effectiveness of an anti-bullying program in terms of both victim and bystander’s self-efficacy. That said, there are some studies focused solely on either bystander’s self-efficacy (Hallford et al., 2006; Knauf et al., 2018; Salmivalli et al., 2005; Slee & Mohyla, 2007; Stevens et al., 2004; Thornberg & Jungert, 2013; Thornberg et al., 2017) or victim’s self-efficacy (Salimi et al., 2021). All these studies are also limited in their measurement scales as they do not measure the five steps as indicators of self-efficacy. This limitation calls for the development and validation of new scales that allow for measurement of the five steps of victim and bystander’s self-efficacy in tackling both offline and online bullying. Therefore, the current paper aims to close this gap by developing and testing validity of four new scales related to victim and bystander’s self-efficacy in dealing with bullying behaviors.

## Methods

### Procedures

This work forms part of a larger research project implementing an anti-bullying program in post-primary schools in Ireland. The results and outlines of the wider program are currently in progress and are not being presented in this manuscript. The program was offered to all post-primary schools (*N* = 730) in the country and 121 expressed interest to implement it (March 2020 to June 2021). However, only 41 schools fully completed the program. School principals invited students to complete an online survey about their self-efficacy in tackling offline and online bullying after the implementation of the program in Spring 2021. Students and their parents received an Email with the survey link along with instructions and consent forms. Parents and students were asked to watch a video describing the aim, benefit and process of the survey. Both parents and their children were asked for their consent to participate in the survey. Ethical approval was obtained by the ethics committee of the authors’ university before the distribution of the survey and the program implementation.

### Population and Sample Size

The research involved two different samples for testing content and construct validity of the scales. The content validity test was based on responses of an initial sample of 30 post-primary school students (14-year-old) in Ireland and seven academic researchers, whereas the construct validity test was based on a convenience sample of 1,254 post-primary school students (14-year-old) in Ireland. However, a total of 154 cases were excluded due to random missing values. Therefore, the final sample was 1,100. Given that the research is not focused on individual differences or cross-group comparisons, this section included no descriptive statistics of demographic variables.

### Measures

#### Victim and Bystander’s Self-Efficacy Scales

To measure victim and bystander’s self-efficacy in tackling both offline and online bullying, the present research developed and tested four separate scales below (see Supplemental Appendix for the whole scale and its design):

- Dublin Anti-Bullying Offline Victim’s Self-Efficacy Scale
- Dublin Anti-Bullying Online Victim’s Self-Efficacy Scale
- Dublin Anti-Bullying Offline Bystander’s Self-Efficacy Scale
- Dublin Anti-Bullying Online Bystander’s Self-Efficacy Scale

The design of the scales was adopted from Kuldas (2018) and was based on the five steps of the social-ecological approach and model of anti-bullying self-efficacy for measuring both victim and bystander’s self-efficacy, operationalized as confidence in their ability for the *recognition* (5-item), *emergency comprehension* (5-item), *responsibility* (5-item), *knowledge* (6-item), and *intervention* (5-item). Each step started with the statement “*The Anti-Bullying programme has increased my confidence in my ability. . .*” to recognize bullying behavior, to comprehend emergency for intervention, to take responsibility, to know what to do, and to intervene (a) if I am bullied in person, (b) if I am bullied online, (c) if someone else is bullied in person, and (d) if someone else is bullied online. Students rated their confidence levels for each separate step on a 6-point scale (from 5 = Very to 0 = Not at all).

### Data Analysis

#### Content and Face Validity

A widely held consensus on content and face validity recommends both participants and experts’ involvement in the development of scale items (Kuldas, 2018). Following this recommendation, an initial number of 26 items for each scale were derived from verbal feedback of students who participated in the anti-bullying program. They verbally provided feedback at the end of the program about whether it increased their confidence in tackling bullying behavior. From their comments, three of the authors selected keywords and phrases to form the 26 items in line with the theoretical model. Examples of keywords and phrases are: noticing, realizing, becoming aware, knowing how to tackle bullying, asking for help, understanding emergency, telling an adult, speaking out, taking responsibility, and reporting. Thereafter, a convenience sample of adolescent students (*N* = 30, aged 14 years) who participated in the program was provided with an online survey to rate their levels of agreement on each of the 26-item according to the following seven aspects: this statement/item is (a) clear to me, (b) relevant to me, (c) understandable to me, (d) the instructions on how to respond each item are clear to me, (e) the font size is readable to me, (f) I understand that I am asked to rate my responses separately in the four columns, and (g) the rating scale from 5 (Very) to 0 (Not at all) for each item is clear to me. They ranked their agreement on a 4-point scale (0 = Disagree a lot, 1 = Disagree, 2 = Agree, 3 = Agree a lot). The first two values were coded as 0 (No agreement), while the last two rating values were coded as 1 (Agreement).

Next, to revise the statements in terms of content and dimensions of self-efficacy beliefs, 11 academic researchers from different universities in four different countries were invited via Email. Seven experts in total consented and evaluated the scale content. Regarding their expertise levels, one had five years, three had 11 years, and the rest had more than 20 years of interdisciplinary research experience in bullying behaviors, online safety/risks, the Internet use, and psychometric assessment and evaluation.

An online survey including the original scale was administered to the experts alongside the conceptual definitions of the measured concept and the model used to develop the items, nine questions about (a) the conceptual representativeness (content validity) and (b) the clarity and brevity of items, instructions, and designs (face validity), and instructions on how to rate their agreement. The experts reported their agreement levels for the content validity based on two questions: whether (a) each of the items is representative of the corresponding construct, and (b) the concept/label represents its group of items. An accurate judgment of content validity can be done based on these two questions (MacKenzie et al., 2011). For the face validity, they reported their levels of agreement on seven aspects, whether items are (a) clear, (b) concise, (c) free from grammatical mistakes, (d) free from spelling errors, (e) understandable to 13- to 18-year-old students in Ireland, also whether (f) instructions and (g) the survey design are clear for rating each item. Each expert had to rate each item of the original scale on a 4-point scale (0 = Not accepted as it is, 1 = Not accepted without major revisions, 2 = Accepted with minor revisions, 3 = Accepted as it is). All their answers were coded as 0 = No agreement and 1 = Agreement. The rating values of 0 and 1 for the original items on the nine questions were coded as 0 (No agreement), while the rating values of 2 and 3 were coded as 1 (Agreement). In cases where the raters required minor or major revisions, a comment box was available to write their recommendations.

Agreement levels of both students and experts can be calculated with regard to Item-Content Validity Index (I-CVI) and Scale-Content Validity Index (S-CVI), respectively estimating the content representativeness of each item and group of items (Polit & Beck, 2006). To estimate I-CVI, the number of students/experts who rated as agreed on each item was divided by the total number of students/experts (Polit & Beck, 2006). The sum of agreement on the I-CVI was then divided by the total number of items to obtain S-CVI (Polit & Beck, 2006). Among a panel of five raters, the agreement on one item must be 100%, but with six or more raters, ≥80% of agreement is sufficient for items to be considered theoretically representative of a targeted construct (Sangoseni et al., 2013).

#### Reliability and Construct Validity

Reliability and construct validity of *Dublin Anti-Bullying Self-Efficacy Scales* were estimated in three steps. First, factorial structures were estimated via four separate exploratory factor analyses (Principal Axis Factoring with Promax Oblique rotation method in IBM SPSS v.27 statistical software) of responses by *offline victims* (26-item), *online victims* (26-item), *offline bystanders* (26-item), and *online bystanders* (26-item). Next, convergent validity (Average Variance Extracted—AVE > 0.50) and discriminant validity (the square root of AVE greater than all the inter-factor correlations, Fornell & Lacker, 1981) were estimated as the criterion for construct validity (Hair et al., 2014). Last, composite reliability was estimated as a measure of internal consistency (Hair et al., 2014). These three steps can be considered as testing a reflective measurement model (i.e., testing relationships between a latent construct and its reflective indicators), which is evaluated in terms of internal consistency, convergent validity, and discriminant validity (Lewis et al., 2005).

## Results

### Content and Face Validity

The estimated content and face validity indices indicated 96% agreement among the students. However, reclassification of items on the basis of the two questions were iterated two times by all the experts to reach a common agreement on the content and face validity of each construct. The calculated indices indicated ≥91% of the experts’ agreement on five main groups of items reflecting the five steps, labelled and defined as follow:

- First, *recognition* of bullying behavior is defined as the victim/bystander’s confidence in their ability to notice, to be aware, or to realize what bullying behavior is (if they are or someone else is bullied).
- Second, *emergency comprehension* is defined as the victim/bystander’s confidence in their ability to comprehend or realize the need for urgent help, asking for help, taking action, or telling someone as an intervention in bullying behavior.
- Third, *responsibility* is defined as the victim/bystander’s confidence in their ability to take personal responsibility for reporting, telling someone, speaking out, or taking action against bullying behavior.
- Fourth, *knowledge* is defined as the victim/bystander’s confidence in their ability to know what to do, how to report, where to report, or whom to ask for help against bullying behavior.
- Fifth, *intervention* is defined as the victim/bystander’s confidence in their ability to report, to tell someone, or to ask for help against bullying behavior.

#### Reliability and Construct Validity

Assumptions for conducting an Exploratory Factor Analysis (EFA) were tested and satisfied. The inter-item correlation index of each scale was screened to detect if there was an issue of multicollinearity. The matrix displayed that only 20 out of the 26 items of each scale had no inter-item correlation value exceeding >.80, which indicated no multicollinearity (Pett et al., 2003). Therefore, a total of six items from each scale were removed from further analysis. The KMO measure = 0.93, 0.93, 0.93, and 0.92 along with significant Bartlet test (*p* < .001) verified the sampling adequacy for the EFA of the (a) victim’s self-efficacy scale in bullying offline, (b) victim’s self-efficacy scale in bullying online, (c) bystander’s self-efficacy scale in bullying offline, and (d) bystander’s self-efficacy scale in bullying online, respectively. A five-factor solution, from each and every scale with 20 items, had eigenvalues over Kaiser’s criterion of 1. Composite Reliability (CR > 0.70), convergent validity (AVE > 0.50), and discriminant validity (the square root of AVE > the inter-factor correlations, Fornell & Lacker, 1981) satisfied the criterion for reliability and construct validity for all the four scales (Hair et al., 2014). Table 1 displays rotated factor loadings of 20 items of each scale as well as the estimated values for composite reliability, convergent validity, and discriminant validity.

**Table 1.** Results of Four Separate Exploratory Factor Analyses of the Dublin Anti-Bullying Self-Efficacy Scales.

Item	if I am bullied in person (Offline Victims)					if I am bullied online (Online Victims)					if someone else is bullied in person (Offline Bystanders)					if someone else is bullied online (Online Bystanders)				
	F1	F2	F3	F4	F5	F1	F2	F3	F4	F5	F1	F2	F3	F4	F5	F1	F2	F3	F4	F5
Recognition																				
to be aware	0.913					0.943					0.904							0.909		
to realise	0.896					0.886					0.914							0.920		
to notice	0.879					0.912					0.891							0.888		
to recognise bullying behaviours	0.816					0.767					0.796							0.784		
Emergency Comprehension																				
to see the need to ask for help			0.950					0.944							0.895				0.917	
to see the need to tell someone			0.862					0.837							0.848				0.863	
to see the need for urgent help			0.836					0.859							0.840				0.858	
to see the need to take action			0.678					0.724							0.814				0.872	
Responsibility																				
to take responsibility for speaking out				0.937					0.866				0.909			0.925				
to take responsibility for reporting				0.866					0.826				0.856			0.906				
to take responsibility for telling someone				0.806					0.868				0.857			0.876				
to take responsibility for taking action				0.761					0.753				0.846			0.856				
Knowledge																				
to know where to report		0.885					0.903							0.851						0.884
to know what to do		0.837					0.861							0.877						0.855
to know whom to ask for help		0.762					0.724							0.836						0.798
to know how to report		0.744					0.742							0.817						0.808
Intervention																				
to tell someone					0.913					0.826		0.892					0.939			
to ask for help					0.891					0.892		0.897					0.929			
to report					0.746					0.744		0.860					0.825			
where to report					0.683					0.633		0.797					0.807			
Eigenvalue	9.36	2.84	1.60	1.15	1.02	9.10	2.91	1.79	1.10	1.01	9.06	2.92	1.93	1.26	1.91	8.75	2.68	2.05	1.74	1.33
% of Variance	45.5	12.9	6.8	4.4	3.8	44.2	13.3	7.7	4.2	3.6	44.1	13.4	8.5	5.1	4.7	42.6	12.3	9.2	7.6	5.4
Convergent Validity	0.77	0.65	0.70	0.71	0.66	0.77	0.66	0.71	0.69	0.61	0.77	0.74	0.75	0.71	0.72	0.79	0.77	0.77	0.77	0.70
Discriminant Validity	0.88	0.81	0.84	0.85	0.81	0.88	0.81	0.84	0.83	0.78	0.88	0.86	0.87	0.85	0.85	0.89	0.88	0.88	0.88	0.84
Composite Reliability	0.93	0.88	0.90	0.91	0.89	0.93	0.88	0.91	0.90	0.86	0.93	0.92	0.92	0.91	0.91	0.94	0.93	0.93	0.93	0.90

*Note. N* = 1,100. The extraction method was Principal Axis Factoring with Promax Oblique Rotation with Kaiser normalization. All factor loadings were well above 0.32. Convergent validity was estimated via Average Variance Extracted (AVE = the sum of the squared loadings divided by the number of indicators). Discriminant validity was estimated via the square root of AVE greater than inter-factor correlations.

## Discussion

Effectiveness of anti-bullying programs is generally assessed in terms of their contributions to students’ awareness, implementations of school anti-bullying policy, and promotion of positive school climate (Foody et al., 2018). Such an assessment method lacks a central focus on the development and measurement of anti-bullying self-efficacy beliefs, which play a central role in the prevention and/or intervention of offline and online bullying (Salimi et al., 2021). Effectiveness of anti-bullying programs also depends on the extent to which victim and bystander’s self-efficacy beliefs are developed and measured (Green et al., 2020; Nocentini & Menesini, 2016; O’Moore & Minton, 2005; Salimi et al., 2021; Thornberg et al., 2017). However, the extent to which this aim is achieved has yet to be accurately assessed, mainly due to the absence of scales measuring effectiveness of an anti-bullying program in terms of victim and bystander’s anti-bullying self-efficacy. To facilitate further research on the accurate assessment of effectiveness of anti-bullying programs, the present study has taken four main steps to identify and measure various dimensions of anti-bullying offline and online self-efficacy beliefs among both victims and bystanders.

The first step was a critical review of the traditional and revised definitions of bullying and cyberbullying, so as to determine the extent to which the online and offline phenomena have distinct or common characteristics. According to the new definition of school bullying by UNESCO (2020), the criteria of repetitiveness and intentionality for the traditional definition are no longer applicable. UNESCO has hereby advocated the argument for the overlap between offline and online bullying characteristics, as they both require a social network, harmful effect, and an imbalance of power in order to be defined. However, the proposed overlap does not necessarily mean that self-efficacy beliefs in tackling online and offline bullying also overlap. For example, self-efficacy in tackling a physical form of offline bullying can vary depending on a power-imbalance, which is different when tackling online bullying (e.g., digital skills). Such differences warrant separately measuring anti-bullying online and offline self-efficacy. The current research has therefore developed and tested four *separate* anti-bullying scales for measuring: (1) offline victim’s self-efficacy, (2) online victim’s self-efficacy, (3) offline bystander’s self-efficacy, and (4) online bystanders’ self-efficacy.

The second step was aimed at identifying various dimensions of anti-bullying self-efficacy beliefs. This identification was achieved through an integrative review of the participant role approach focusing on the following first three steps and bystander intervention model suggesting the rest five steps: (1) *raising awareness*, (2) *self-reflection*, (3) *commitment to anti-bullying behaviors* (Salmivalli et al., 2005), (4) *noticing* the event, (5) *interpreting* it as emergency, (6) taking or accepting personal *responsibility* to act (Latané & Darley, 1970), (7) choosing an appropriate option to intervene, and (8) implementing a way of intervention (Knauf et al., 2018; Nickerson et al., 2014).

The third step was aimed at synthesizing the participant role approach (Salmivalli et al., 2005) and bystander intervention model (Latané & Darley, 1970). Based on this synthesis, the present research proposed *a Social-Ecological Approach and Model of Anti-Bullying Self-Efficacy* by regrouping the eight overlapping steps into the five sequential steps that victims and bystanders take to intervene in online and offline bullying behaviors. The proposed approach hereby suggests considering anti-bullying self-efficacy as a developmental capacity, process, and outcome of person-environment interactions within an intersectional context. For instance, a caring and supportive teacher, parent, and/or friend can help victims develop and demonstrate self-efficacy against bullying (Kuldas & Foody, 2022) in each step. However, the development or promotion of one step is not enough (Nickerson et al., 2014). For example, victim and bystander’s awareness of bullying or the need to stop it is not sufficient for the intervention to take place (Wachs et al., 2018). Effectiveness of anti-bullying programs requires the promotion of anti-bullying self-efficacy in each step. In other words, effectiveness of an anti-bullying program would be more accurately assessed when each step of anti-bullying self-efficacy is taken into account.

The final step was an initial derivation of all the scale items from comments of adolescent students (i.e., participants involved in the item formation). Thereafter, a group of seven academic researchers established the content and face validity. Next, four separate exploratory factor analyses (i.e., estimating number of retainable factors) and reflective measurement model analyses (i.e., estimating convergent and discriminant validity) identified sufficient psychometric properties (reliability and construct validity) of the four *separate* Dublin Anti-Bullying Self-Efficacy Scales. Each scale with 20 items separately had a five-factor solution. The five factors are labelled as *recognition, emergency comprehension, responsibility, knowledge*, and *intervention.* This result suggests that the newly developed scales can adequately assess adolescents’ anti-bullying self-efficacy beliefs in tackling both offline and online bullying situations as both victims and bystanders. This novel finding in itself provides an important step forward to the anti-bullying literature as it allows researchers to measure anti-bullying self-efficacy beliefs that might occur as a result of the entire anti-bullying programs.

### Limitations and Future Research

The present research has some theoretical and methodological limitations and delimitations that restrict the generalization and implications of the findings. The main limitations include the use of a convenience sampling method, only exploratory factor analyses (i.e., no test of structural model or measurement invariance addressing issues of diversity), and the term victim.

First, although the anti-bullying program was offered nationally to all schools in Ireland, only a specific number decided to participate. Therefore, the findings cannot be generalized to the national population.

Next, only convergent and discriminant validity of the four reflective measurement scales were tested. The research therefore included no test of measurement invariance by age, sex, ethnicity, sexual orientation, religion, and/or socioeconomic status groups. The lack of measurement invariance test does not allow to make empirical conclusions about how diversity affects the scale development and the creation of an effective intervention program. Therefore, the research failed to address issues in diversity, leaving unclear whether the anti-bullying self-efficacy scales allow for a group comparison. To address this issue, a Confirmatory Factor Analysis (CFA) of the structural model and measurement invariance is needed. To perform a CFA, a different sample is required (Costello & Osborne, 2005). It should also be noted that assumptions for conducting further EFA or CFA of the scales should be tested, especially outliers. Otherwise, further analysis might not yield the five-factor solution. In other words, the five-factor solution requires future research to meet all the assumptions (e.g., excluding outliers from data analysis).

Last, the term “victim” was used throughout this manuscript and in the scales developed therein to be consistent with the synthesized approach and model. However, the discourse in the various fields of bullying research over the last decade has shifted away from the use of the terms bully and victim to more empowering terms perpetrator and target (Branch et al., 2021). In particular, to replace the term victim with *target* is suggested because it may help detract from the notion of “helpless victim” and “victim-blaming attitude” that implies if the person had behaved differently, he or she might have been able to prevent the proactive aggressive behavior and its consequences (McLeer, 1998). Therefore, the term target can be used with the aim to help adolescents and youth feel empowered and they can respond effectively without considering themselves as the helpless victim (Willard, 2007). Such a use of the term is in line with the aim to measure effectiveness of anti-bullying intervention program in terms of promoting victim’s self-efficacy.

Despite these limitations, the present research has provided preliminary psychometric evidence for face, content, construct validity, and reliability. It hereby proposed anti-bullying self-efficacy models and scales, a novel way to measure effectiveness of anti-bullying programs. The five dimensions of the proposed model are applicable to both victims and bystanders of either offline or online bullying, yet differences in their self-efficacy were not tested. Future research is recommended to test whether (a) there are significant differences between victims and bystander’s self-efficacy and/or (b) self-efficacy in tackling offline bullying differs from online bullying. Future research might also need to test the sequence of the five steps, which one precedes and/or follows.

## Conclusions

Anti-bullying programs are mostly focused on the rise or fall of bullying and victimization rates as their measure of effectiveness. Such a measurement does not allow for assessing effectiveness of the entire program in making changes in the cognitive aspect, namely anti-bullying self-efficacy beliefs. This lack of assessment leaves no empirical evidence for the growing consensus that anti-bullying programs should also focus on promoting anti-bullying self-efficacy beliefs among victims and bystanders. However, current literature on anti-bullying programs falls short in addressing this need for empirical evidence, mainly due to theoretical and empirical gaps. There was (a) no theoretical approach and model that explains dimensions of anti-bullying self-efficacy beliefs among both victims and bystanders, and (b) no scale that measures dimensions of self-efficacy beliefs, the extent to which victims and bystanders have confidence in their ability for tackling online and offline bullying.

Therefore, novelty of the current research has manifested itself in filling both theoretical and empirical gaps by the proposed (a) *Social-Ecological Approach and Model of Anti-Bullying Self-Efficacy* and (b) *Dublin Anti-Bullying Self-Efficacy Scales*, whereby were identified the five dimensions of self-efficacy beliefs, namely recognition, emergency comprehension, responsibility, knowledge, and intervention. The research provided preliminary evidence for the sufficiency of psychometric properties of the four scales measuring both victim and bystander’s self-efficacy beliefs in tackling both online and offline bullying situations. As the main implications, the proposed approach, model, and scales can allow further research to adequately assess adolescents’ anti-bullying self-efficacy beliefs in tackling both offline and online bullying situations as both victims and bystanders. The research hereby provides theoretical and empirical steps forward in the anti-bullying literature as it allows measuring cognitive changes that might occur as a result of the entire anti-bullying programs, which might otherwise have been overlooked.

## Supplemental Material

sj-docx-1-jiv-10.1177_08862605221127193 – Supplemental material for Dublin Anti-Bullying Self-Efficacy Models and Scales: Development and Validation Click here for additional data file. Supplemental material, sj-docx-1-jiv-10.1177_08862605221127193 for Dublin Anti-Bullying Self-Efficacy Models and Scales: Development and Validation by Aikaterini Sargioti, Seffetullah Kuldas, Mairéad Foody, Paloma Viejo Otero, Angela Kinahan, Colm Canning, Darran Heaney and James O’Higgins Norman in Journal of Interpersonal Violence

## Appendix

**Appendix.** Dublin Anti-Bullying Self-Efficacy Scales with the initial 26-Item.

Mark the following statements from 5 = Very to 0 = Not at all		**Very**				
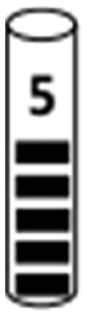
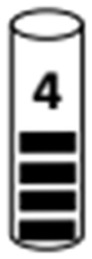
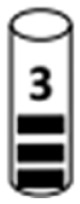
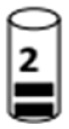
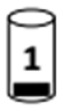
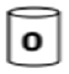
			If I am bullied		If someone else is bullied	
**Dimensions**	**Victim and Bystanders’ Self-efficacy**		**in person**	**online**	**in person**	**online**
**Recognition**	The Anti-Bullying programme has increased my confidence in my ability. . .	to notice				
		to be aware				
		to realise				
		to recognise bullying behaviours				
		to identify the bully*				
**Emergency Comprehension**	The Anti-Bullying programme has increased my confidence in my ability . . .	to see the need to take action				
		to see the need to ask for help				
		to see the need for urgent help				
		to see the need to tell someone				
		to see the need to speak out*				
**Responsibility**	The Anti-Bullying programme has increased my confidence in my ability . . .	to take responsibility for reporting				
		to take responsibility for speaking out				
		to take responsibility for telling someone				
		to take responsibility for taking action				
		to take responsibility for asking for help*				
**Knowledge**	The Anti-Bullying programme has increased my confidence in my ability . . .	to know how to report				
		to know what to do				
		to know where to report				
		to know whom to ask for help				
		to know who to tell*				
		to know why to ask help*				
**Intervention**	The Anti-Bullying programme has increased my confidence in my ability . . .	to report				
		where to report				
		to tell someone				
		to ask for help				
		in what to do*				

*Note*. * = Items with multi-collinearity (exceeding >.80) were removed from the final scale.
