# Supplementary material for: Dublin Anti-Bullying Self-Efficacy Models and Scales: Development and Validation
Source: J Interpers Violence. 2022 Sep 30;38(7-8):5748–73. doi: 10.1177/08862605221127193 (PMC9969485; doi:10.1177/08862605221127193)
Supplement: sj-docx-1-jiv-10.1177_08862605221127193 – Supplemental material for Dublin Anti-Bullying Self-Efficacy Models and Scales: Development and Validation [file sj-docx-1-jiv-10.1177_08862605221127193.docx]

# Appendix

# Dublin Anti-Bullying Self-Efficacy Scales with the initial 26-Item

| \| **Very**  **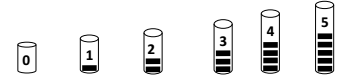** \| **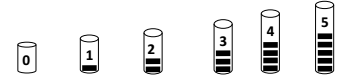** \| **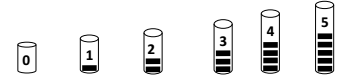** \| **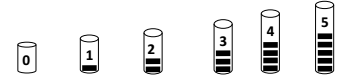** \| **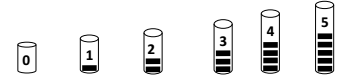** \| **Not**  **at all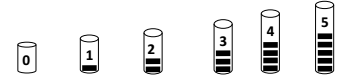** \| \| --- \| --- \| --- \| --- \| --- \| --- \|   Mark the following statements from 5 = Very to 0 = Not at all | | | | | | |
| --- | --- | --- | --- | --- | --- | --- | --- | --- | --- | --- | --- | --- |
|  |  |  | If I am bullied | | If someone else is bullied | |
| **Dimensions** | **Victim and Bystanders’ Self-efficacy** |  | **in person** | **online** | **in person** | **online** |
| **Recognition** | The Anti-Bullying programme has increased my confidence in my ability… | to notice |  |  |  |  |
|  |  | to be aware |  |  |  |  |
|  |  | to realise |  |  |  |  |
|  |  | to recognise bullying behaviours |  |  |  |  |
|  |  | to identify the bully* |  |  |  |  |
|  |  |  |  |  |  |  |
| **Emergency Comprehension** | The Anti-Bullying programme has increased my confidence in my ability … | to see the need to take action |  |  |  |  |
|  |  | to see the need to ask for help |  |  |  |  |
|  |  | to see the need for urgent help |  |  |  |  |
|  |  | to see the need to tell someone |  |  |  |  |
|  |  | to see the need to speak out* |  |  |  |  |
|  |  |  |  |  |  |  |
| **Responsibility** | The Anti-Bullying programme has increased my confidence in my ability … | to take responsibility for reporting |  |  |  |  |
|  |  | to take responsibility for speaking out |  |  |  |  |
|  |  | to take responsibility for telling someone |  |  |  |  |
|  |  | to take responsibility for taking action |  |  |  |  |
|  |  | to take responsibility for asking for help* |  |  |  |  |
|  |  |  |  |  |  |  |
| **Knowledge** | The Anti-Bullying programme has increased my confidence in my ability … | to know how to report |  |  |  |  |
|  |  | to know what to do |  |  |  |  |
|  |  | to know where to report |  |  |  |  |
|  |  | to know whom to ask for help |  |  |  |  |
|  |  | to know who to tell* |  |  |  |  |
|  |  | to know why to ask help* |  |  |  |  |
|  |  |  |  |  |  |  |
| **Intervention** | The Anti-Bullying programme has increased my confidence in my ability … | to report |  |  |  |  |
|  |  | where to report |  |  |  |  |
|  |  | to tell someone |  |  |  |  |
|  |  | to ask for help |  |  |  |  |
|  |  | in what to do* |  |  |  |  |
| *Note*. * = Items with multi-collinearity (exceeding >.80) were removed from the final scale. | | | | | | |
